# Quantitative Analysis of Histone Modifications: Formaldehyde Is a Source of Pathological N^6^-Formyllysine That Is Refractory to Histone Deacetylases

**DOI:** 10.1371/journal.pgen.1003328

**Published:** 2013-02-28

**Authors:** Bahar Edrissi, Koli Taghizadeh, Peter C. Dedon

**Affiliations:** 1 Department of Biological Engineering, Massachusetts Institute of Technology, Cambridge, Massachusetts, United States of America; 2 Center for Environmental Health Sciences, Massachusetts Institute of Technology, Cambridge, Massachusetts, United States of America; The University of North Carolina at Chapel Hill, United States of America

## Abstract

Aberrant protein modifications play an important role in the pathophysiology of many human diseases, in terms of both dysfunction of physiological modifications and the formation of pathological modifications by reaction of proteins with endogenous electrophiles. Recent studies have identified a chemical homolog of lysine acetylation, N^6^-formyllysine, as an abundant modification of histone and chromatin proteins, one possible source of which is the reaction of lysine with 3′-formylphosphate residues from DNA oxidation. Using a new liquid chromatography-coupled to tandem mass spectrometry method to quantify all N^6^-methyl-, -acetyl- and -formyl-lysine modifications, we now report that endogenous formaldehyde is a major source of N^6^-formyllysine and that this adduct is widespread among cellular proteins in all compartments. N^6^-formyllysine was evenly distributed among different classes of histone proteins from human TK6 cells at 1–4 modifications per 10^4^ lysines, which contrasted strongly with lysine acetylation and mono-, di-, and tri-methylation levels of 1.5-380, 5-870, 0-1400, and 0-390 per 10^4^ lysines, respectively. While isotope labeling studies revealed that lysine demethylation is not a source of N^6^-formyllysine in histones, formaldehyde exposure was observed to cause a dose-dependent increase in N^6^-formyllysine, with use of [^13^C,^2^H_2_]-formaldehyde revealing unchanged levels of adducts derived from endogenous sources. Inhibitors of class I and class II histone deacetylases did not affect the levels of N^6^-formyllysine in TK6 cells, and the class III histone deacetylase, SIRT1, had minimal activity (<10%) with a peptide substrate containing the formyl adduct. These data suggest that N^6^-formyllysine is refractory to removal by histone deacetylases, which supports the idea that this abundant protein modification could interfere with normal regulation of gene expression if it arises at conserved sites of physiological protein secondary modification.

## Author Summary

Oxidative stress and inflammation lead to the generation of a multitude of electrophiles in cells that in turn react with nucleophilic macromolecules such as DNA, RNA, polyunsaturated fatty acids, and proteins, leading to progression of a variety of disorders and diseases. Emerging evidence points to widespread modification of cellular proteins by N^6^-formylation of lysine as a result of adventitious reactions with endogenous electrophiles. N^6^-Formyllysine is a chemical homolog of the biologically important N^6^-acetyllysine and thus may interfere with acetylation signaling in cells. While N^6^-formyllysine adducts are now well recognized as abundant protein modifications in cells, the source of these pathological adducts remains unclear. Our previous study proposed N^6^-formylation of lysine in histone proteins occurred by reaction of lysine with 3′-formylphosphate residues arising from DNA oxidation. Here, we investigate additional sources as well as the fate of this abundant pathological protein modification. Our results reveal that endogenous formaldehyde is a major source of N^6^-formyllysine and that this adduct is widely distributed among proteins in all cell compartments. We also demonstrate for the first time that N^6^-formyllysine modifications do not undergo appreciable removal by histone deacetylases, which suggests that they persist in proteins and possibly interfere with the signaling functions at conserved lysine positions in histone proteins.

## Introduction

In addition to physiological secondary modifications, proteins are subjected to reactions with endogenous electrophiles generated by oxidative stress, inflammation, and normal cell metabolic processes [1]–[5]. These adventitious or pathological modifications typically arise by reaction of the nucleophilic side chains of lysine, histidine, and cysteine with reactive electrophiles such as malondialdehyde, 4-hydroxynonenal (HNE), and glyoxal generated by oxidation of polyunsaturated fatty acids and carbohydrates, among other biomolecules [2]–[4], [6], [7]. The resulting adducts, which can alter protein function and lead to protein degradation, are associated with a variety of pathological processes and human diseases [1]–[5], [8]. Among these pathological adducts, N^6^-formylation of lysine has recently emerged as an abundant protein modification [5], [9]–[11]. While originally described in chromatin proteins [9]–[11], it has since been identified as an adduct arising in proteins subjected to nitrosative and oxidative stresses [5]. In chromatin proteins, N^6^-formyllysine has the potential to interfere with the functions of other post-translational modifications that perform signaling functions [12]–[15], such as acetylation, methylation, phosphorylation, ubiquitylation, and ADP ribosylation, with some locations modified in more than one way (*e.g.*, refs. [16]–[18]). The chemical similarities of N^6^-formyllysine and N^6^-acetyllysine suggest a disruptive role for the former in signaling by histone acetylation. Indeed, N^6^-formyllysine has been detected at conserved sites of lysine acetylation and methylation in histones [10], [11].

**Figure 1:**
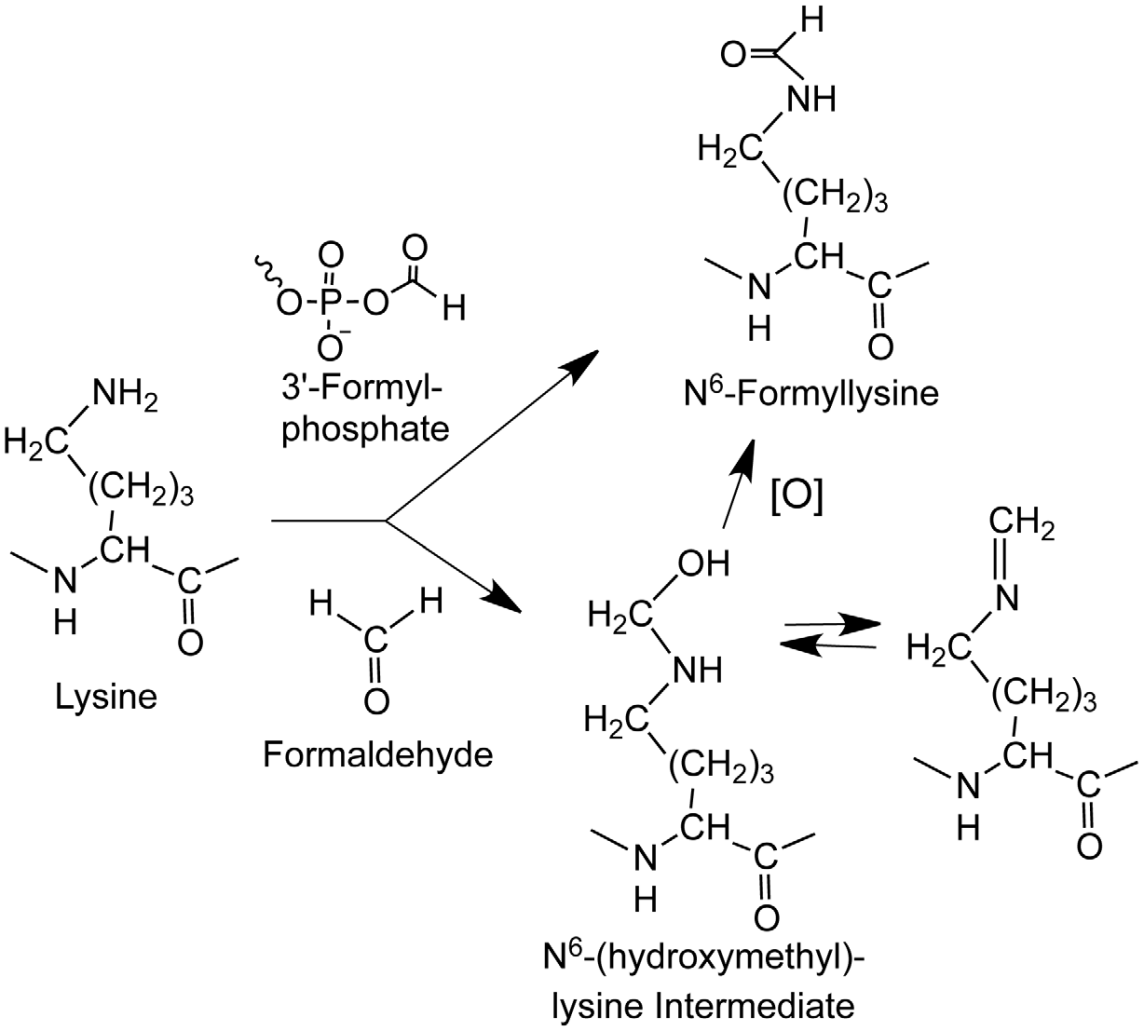
Sources of N^6^-formyllysine. The adduct can be generated in chromatin proteins from reaction of lysine with 3′-formylphosphate residue derived from 5′-oxidation of 2-deoxyribose in DNA or from reaction of lysine with endogenous or exogenous formaldehyde. Formaldehyde reacts with amines to give a carbinolamine intermediate (N^6^-(hydroxymethyl)-lysine) that is in equilibrium with a Schiff base and that is one oxidation state away from the formamide functional group of N^6^-formyllysine.

While N^6^-formyllysine adducts are now well recognized as abundant protein modifications in cells, the source of these pathological adducts remains unclear. We recently showed that some portion of N^6^-formyllysine arises in chromatin proteins by reaction of lysine side chains with the 3′-formylphosphate residue derived from 5′-oxidation of 2-deoxyribose in DNA in cells (Figure 1) [9]. However, the observation of this adduct in proteins treated with the biological oxidant, peroxynitrite, suggests other sources for the formyl species [5]. Considering that formaldehyde reacts with amines to give a carbinolamine intermediate that is only one oxidation state away from a formamide functional group (Figure 1), we hypothesized that endogenous formaldehyde could serve as a source of N^6^-formyllysine residues in histone and other proteins. In addition to environmental and occupational sources [19]–[21], formaldehyde arises from cellular processes such as oxidative demethylation of nucleic acid and histone proteins, as well as biosynthesis of purines, thymidine, and some amino acids [20], [22], [23], making it a relatively abundant metabolite at concentrations ranging from 13 to 97 µM in human plasma [20]. To test this hypothesis and to clarify the cellular locations and quantities of N^6^-formyllysine relative to other major histone modifications, we developed a novel liquid chromatography-coupled electrospray tandem mass spectrometry (LC-MS/MS) method to quantify all N^6^-methyl-, -acetyl-, and -formyl-lysine modifications. Application of this method reveals that endogenous formaldehyde is a major source of N^6^-formyllysine, that this adduct is widespread among proteins in all cellular compartments, and that, in chromatin proteins, it is refractory to removal by histone deacetylases.

## Results

### Quantification of N^6^-lysine modifications in proteins

**Figure 2:**
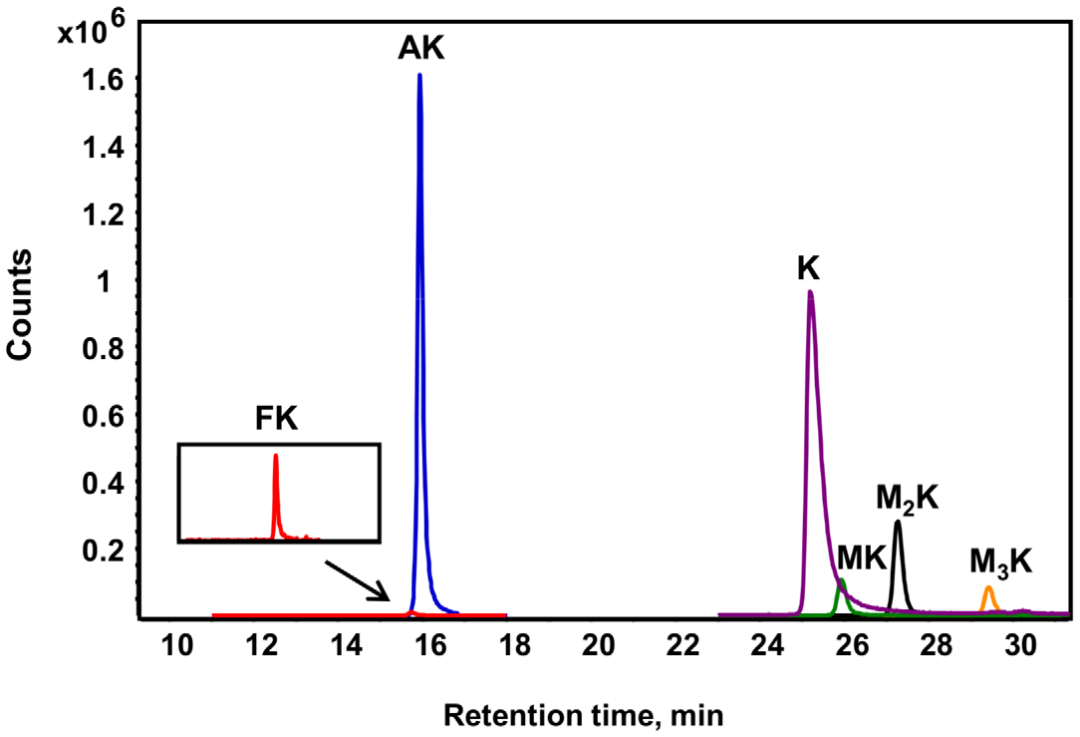
Different lysine species detected in purified histone H4 from TK6 cells. Lysine adducts were monitored by tandem mass spectrometry, as described in *Materials and Methods*. Abbreviations: FK, N^6^-formyllysine; AK, N^6^-acetyllysine; K, lysine; MK, N^6^-mono-methyllysine; M_2_K, N^6^-di-methyllysine; M_3_K, N^6^-tri-methyllysine.

Our previous method for quantifying N^6^-formyllysine in proteins involved proteinase K-mediated hydrolysis of proteins, derivatization of the resulting amino acids with phenylisothiocyanate (PITC), HPLC pre-purification of amino acid derivatives, and final LC-MS/MS analysis of the derivatized amino acids [9]. This method proved to be relatively insensitive and biased as a result of using proteinase K, which produced only partial hydrolysis of some proteins when used in small quantities to minimize background autolysis. To resolve these problems, we used *Streptomyces griseus* protease at ratio of 1 µg enzyme per 10 µg proteins, which resulted in efficient and complete digestion of all proteins as judged by comparing the measured amount of lysine released per µg of purified histone proteins to the theoretical lysine content of the proteins. In addition, the method was optimized to eliminate HPLC pre-purification step, and the need for PITC derivatization to achieve chromatographic resolution of amino acids was obviated by use of aqueous normal phase HPLC with a diamond-hydride column. This chromatographic system resolved N^6^-acetyllysine, mono-, di-, and tri-N^6^-methyllysines, as well as N^6^-formyllysine and lysine, as shown in Figure 2. With isotopically labeled internal standards added prior to protease digestion, identification and quantification of amino acids were accomplished by HPLC-coupled to tandem quadrupole mass spectrometry in positive ion mode, using multiple reaction monitoring (MRM) transitions. With a 2% precision for technical replicates, the limits of detection were found to be 1 fmol for N^6^-formyl- and N^6^-acetyllysine, 10 fmol for lysine, and 50 fmol for each of N^6^-mono-, di-, and tri-methyl lysine. Data for the various lysine modifications are expressed here as proportions of the total number of lysines in the sample.

To validate the new analytical method for lysine modifications, we compared the frequency of N^6^-formyllysine among different classes of histone proteins extracted from TK6 cells and resolved by reversed-phase HPLC. As shown in Figure S1A, all of the major histone classes were separated, with further resolution of variant forms of histones H1 and H3 (SDS-PAGE verification in Figure S1B), which is consistent with previous observations in cultured human cells [10]. N^6^-Formyllysine was detected in all histone classes at a frequency of 1–4 modifications per 10^4^ lysines. This 3- to 4-fold variation among histone classes stands in contrast to the 10- to 100-fold variation in the frequency of other modifications (Table 1). The data in Table 1 represent the first absolute quantification of the various lysine acetyl and methyl modifications in histone proteins, and are consistent with published studies of relative quantities of histone modifications using immunologic and radiolabeling techniques [10], [24]–[26]. Histone modification-based signaling involves the location and number of specific modification targets within a histone protein, as well as the frequency of modification of a target among all copies of a particular histone protein. Our data provide some insight into this issue. For example, we observed low-level acetylation and methylation in histone H1, which is consistent with studies using radiolabeled acetate [24], while this low level of modification maps to specific sites in the globular domain and N-terminal tail of histone H1 [10]. This low-level of acetylation and methylation in histone H1 stands in contrast to the high level of acetylation of H2, H3 and H4 (Table 1), which is again supported by studies using radiolabeled acetate [24].

**Table 1:** Quantification of lysine modifications in HPLC-purified histone proteins.^1^

	Formyl^2^	Acetyl	Methyl	Dimethyl	Trimethyl
**H1 (1)** 1	1.5±0.3^3^	1.6±0.2	8.0±2.0	ND	ND
**H1 (2)** 1	1.2±0.2	1.5±0.3	7.0±2.0	ND	ND
**H2A**	2.7±0.2	26±20	20±8.0	60±30	ND
**H2B**	1.7±0.7	66±20	5.0±7.0	ND	85±30
**H3(1)** 1	3.9±0.5	280±130	680±80	1100±220	220±60
**H3(2)** 1	3.6±1.3	380±100	870±30	1400±270	390±140
**H4**	2.6±0.4	73±60	260±80	740±40	3.0±6.0

1 Classes of histone proteins resolved by reversed-phase HPLC, with putative isoforms denoted in parentheses.

2 Column titles denote different N^6^-modifications of lysine.

3 Data are expressed as modifications per 10^4^ total lysines and represent mean ± SD for 3 biological replicates.

The new analytical method was next applied to quantify N^6^-formyllysine in non-histone proteins. We had previously observed N^6^-formyllysine mainly in histone proteins [9], perhaps as a result of biased proteolysis or subsequent steps in the technique. However, using the new method, we are now able to detect N^6^-formyllysine modifications in a variety of different proteins, as shown in Table 2. Further, an analysis of proteins in nuclear, cytosolic, and membrane compartments in bovine liver revealed the presence of N^6^-formyllysine in all three locations (Table 2). These observations are consistent with a source for N^6^-formyllysine other than the 3′-formylphosphate residues of DNA oxidation previously identified for histone proteins [9].

**Table 2:** Quantification of N^6^-formyllysine in different proteins.

Identity of Protein	N^6^-Formyllysine per 10^4^Lys
BSA	5±0.5
IgG	2±0.4
Collagen	2±0.5
HMG-1	<0.2±0.04
Bovine liver nuclear proteins	2±0.6
Bovine liver membrane proteins	4±1.0
Bovine liver cytosolic proteins	4±0.6

### Formaldehyde as a source of N^6^-formyllysine

**Figure 3:**
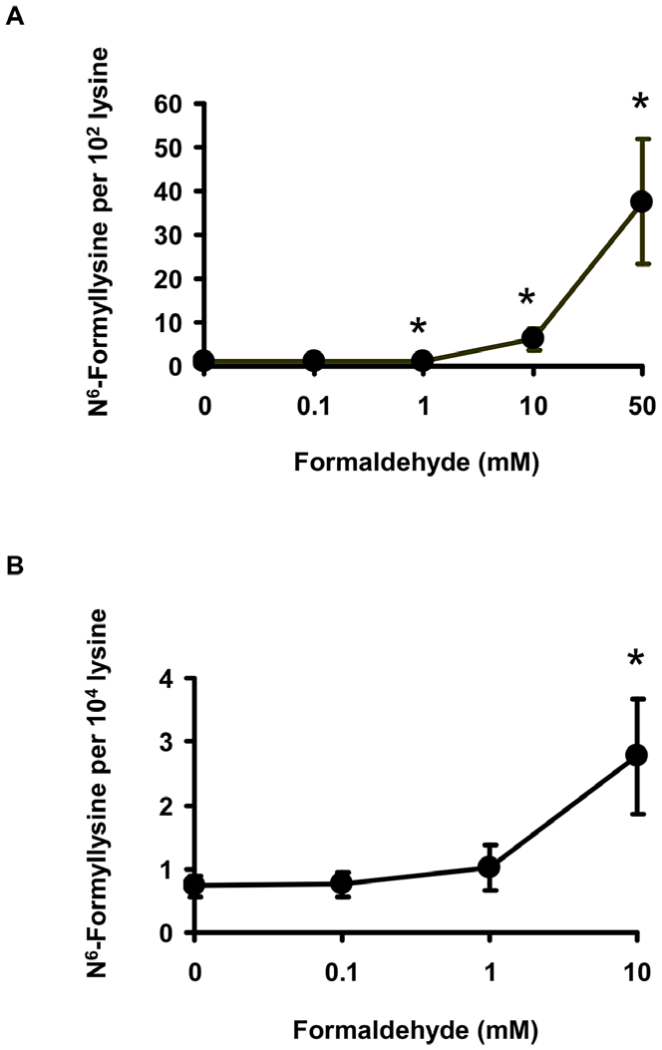
Formaldehyde is a source of N^6^-formyllysine. Formation of N^6^-formyllysine in (A) *in vitro* reactions of 1 mM L-lysine with formaldehyde for 2 h at 37°C, and in (B) TK6 cells exposed to formaldehyde, as described in *Materials and Methods*. Data represent mean ± SD for N = 3, with asterisks denoting statistically significant differences by Student's t-test (p<0.05).

One alternative to 3′-formylphosphate residues as a source of N^6^-formyllysine is oxidation of the carbinolamine intermediate in the reaction of formaldehyde with side chain amine of lysine (Figure 1; N^6^-(hydroxymethyl)-lysine). To test this hypothesis, we performed a series of experiments, starting with an *in vitro* reaction of L-lysine with different concentrations of formaldehyde and quantification of N^6^-formyllysine. As shown in Figure 3A, there was a concentration-dependent formation of N^6^-formyllysine in reactions with formaldehyde, presumably as a result of oxidation of the carbinolamine adduct by the background of reactive oxygen species generated by trace metals and dissolved oxygen in the solution. The oxygen dependence of formaldehyde-induced N^6^-formyllysine was verified by bubbling 100% oxygen (4 h) into the solution of 1 mM lysine and 10 mM formaldehyde, which caused a 2.2 (±0.4)-fold increase in the level of N^6^-formyllysine after 12 h of incubation at 37°C. The dose-response relationship for formaldehyde-induced N^6^-formyllysine formation was also observed in histone proteins extracted from TK6 cells exposed to formaldehyde for 2 h at 37°C (Figure 3B), with 10 mM formaldehyde producing roughly the same fold-change of N^6^-formyllysine in both *in vitro* and cellular studies.

**Figure 4:**
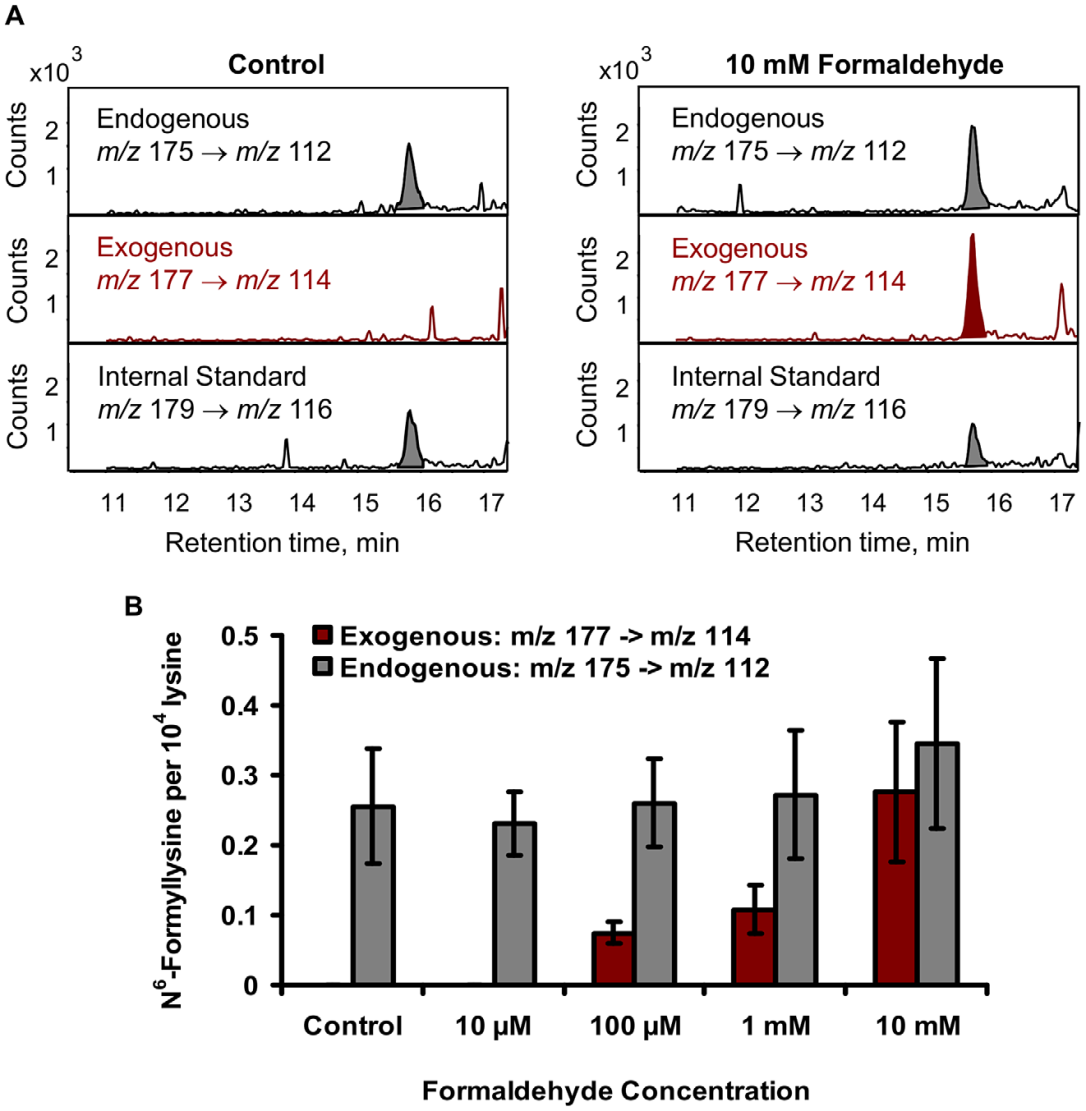
Addition of [^13^C,^2^H_2_]-formaldehyde to TK6 cells distinguishes exogenous from endogenous sources of N^6^-formyllysine. (A) LC-MS/MS analysis showing signals for the three isotopomeric N^6^-formyllysine species, as described in *Materials and Methods*. (B) Plot of N^6^-formyllysine levels as a function of exposure to [^13^C,^2^H_2_]-formaldehyde. Data represent mean ± SD for N = 3.

The relatively high endogenous levels of N^6^-formyllysine in histone and other proteins (Table 1 and Table 2) raised the question of the contribution of exogenous formaldehyde exposures to the total load of N^6^-formyllysine in the cells. To address this issue, we exposed TK6 cells to [^13^C,^2^H_2_]-labeled formaldehyde, which led to the formation of N^6^-[^13^C,^2^H]-formyllysine that is 2 mass units heavier than the endogenous adducts (Figure 4A). Following extraction of the histone proteins from formaldehyde-treated TK6 cells (2 h, 37°C), both endogenous and exogenous N^6^-formyllysine were quantified by monitoring the transitions *m/z* 175→112 and *m/z* 177→114, respectively (Figure 4A), with a third transition (*m/z* 179→116) for the 4,4,5,5-[^2^H]-N^6^-formyllysine internal standard. As shown in Figure 4B, levels of endogenous (unlabeled) N^6^-formyllysine remained constant at all concentrations of [^13^C,^2^H_2_]-formaldehyde, while N^6^-[^13^C,^2^H]-formyllysine increased as a function of the concentration of labeled formaldehyde.

### Lysine demethylation as a source of N^6^-formyllysine

**Figure 5:**
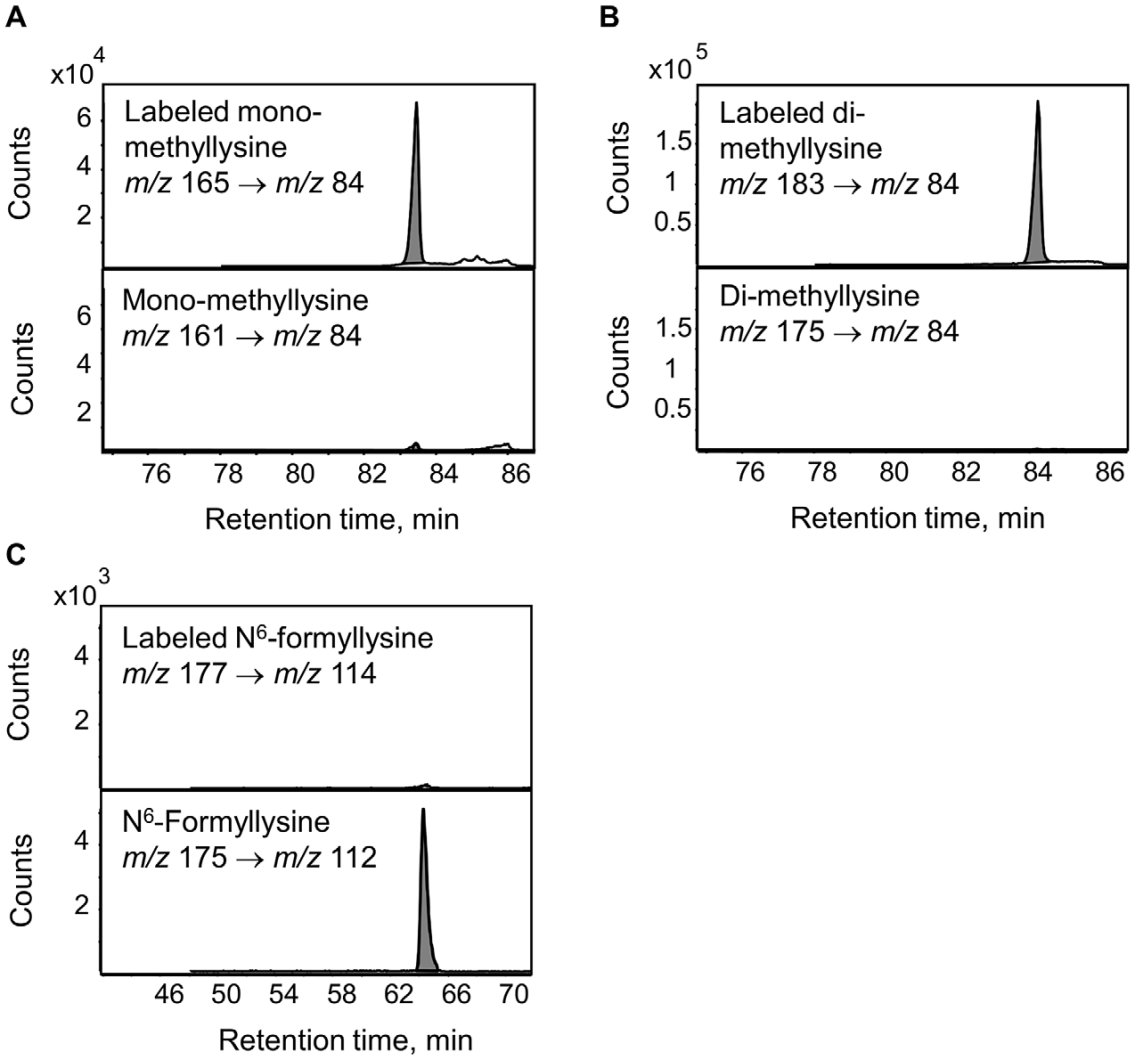
Analysis of lysine demethylation as a source of N^6^-formyllysine. Methyl groups in N^6^-methyllysine species in TK6 cells were labeled using L-methionine-([^13^C,^2^H_3_]-methyl) and N^6^-formyllysine and N^6^-methyllysine species were quantified by LC-MS/MS as described in *Materials and Methods*. Panels A and B: N^6^-mono-methyllysine and N^6^-di-methyllysine are predominately labeled (>90%) with heavy isotope methyl groups (mass increase of 4 *m/z* and 8 *m/z*, respectively), with <10% of the modifications containing unlabeled methyl groups. Panel C: the level of N^6^-[^13^C, ^2^H]-formyllysine (177 *m/z*→114 *m/z* transition) in histones did not show an increase beyond the natural isotope abundance level of ∼0.7% for [M+2] ion of N^6^-formyllysine.

The enzymatic demethylation of N^6^-methyllysine modifications represents another possible source of N^6^-formyllysine in histone proteins, given both the carbinolamine intermediate known to form during the process of lysine demethylation and the ultimate release of the methyl group as formaldehyde [23]. Adventitious oxidation of the carbinolamine intermediate or secondary reaction of the released formaldehyde could result in the formation of N^6^-formyllysine locally. To test these hypotheses, TK6 cells were grown in customized RPMI medium containing L-methionine with a [^13^C,^2^H_3_]-methyl group for 20 days to label all methyl groups in N^6^-methyllysine species, and histone proteins were extracted for analysis every 2 days. If N^6^-formyllysine is a product of disrupted lysine demethylation in histones and is formed via oxidation of the carbinolamine intermediate known to form during the process of lysine demethylation [23], or by reaction of lysine with the formaldehyde released at the last step of successful lysine demethylation [23], then one would expect to see an increase of 2 mass units corresponding to formation of N^6^-[^13^C, ^2^H]-formyllysine (*m/z* 177→114 transition). In order to increase the signal-to-noise ratio for N^6^-[^13^C,^2^H]-formyllysine, N^6^-formyllysine was HPLC-pre-purified in all samples before LC-MS/MS analysis. Figure 5 depicts an example of the analysis using the day 6 sample. As shown in Figure 5, N^6^-mono-methyllysine and N^6^-di-methyllysine are predominately labeled (>90%) with heavy isotope methyl groups (*i.e.*,[^13^C,^2^H_3_]-methyl). In contrast to methyllysines, the level of N^6^-[^13^C,^2^H]-formyllysine did not increase beyond the natural isotope abundance level of ∼0.7% for the [M+2] ion of N^6^-formyllysine (Figure 5C and Figure S2). Note that the HPLC gradient was changed here to fully resolve a contaminant signal from the TK6 cells (identified as the [M+1] ion of citrulline) that otherwise co-eluted with N^6^-formyllysine and produced an *m/z* value similar to the [M+2] isotopomer of N^6^-formyllysine.

### Persistence of N^6^-formyllysine in cells

**Figure 6:**
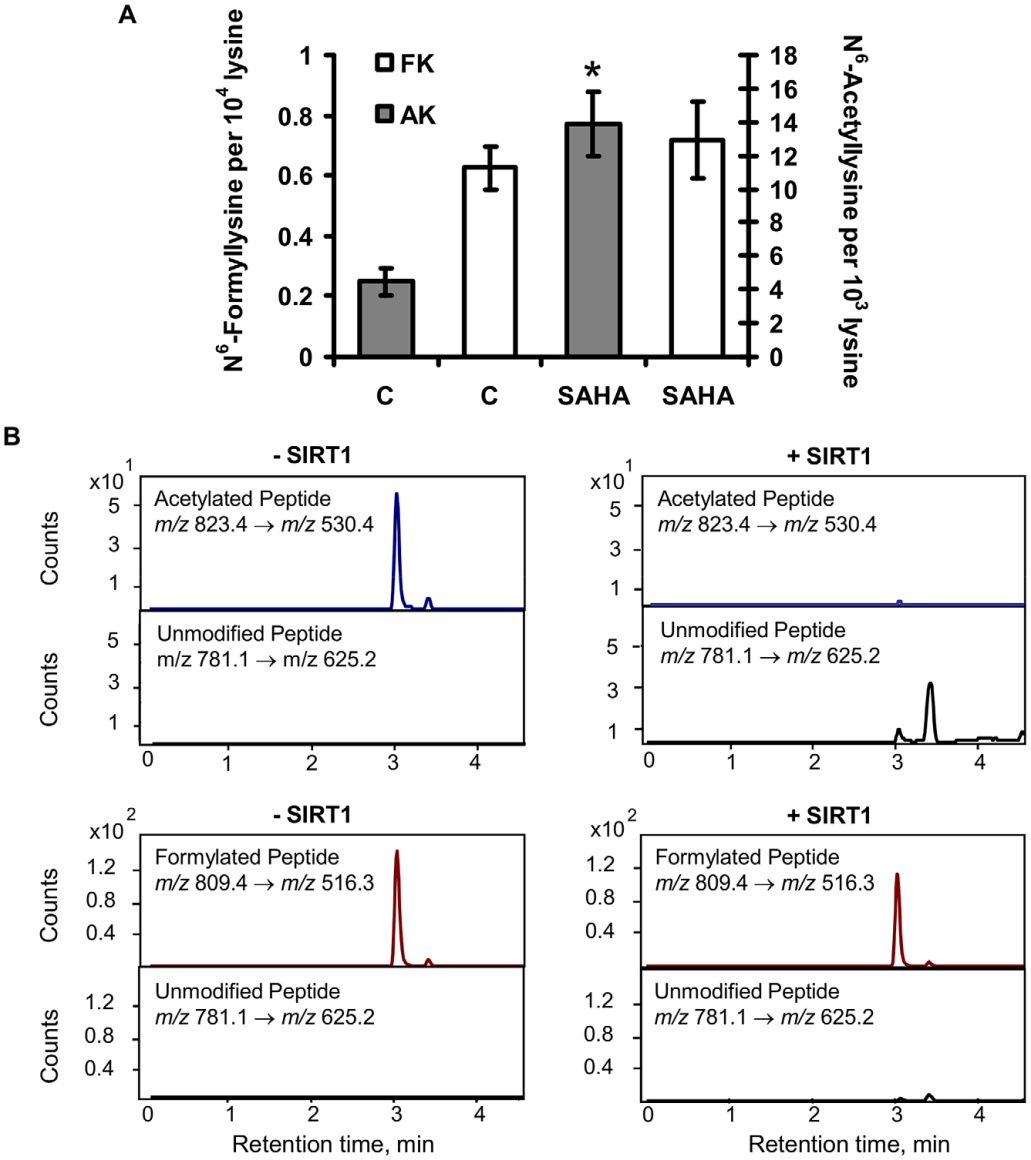
Effect of lysine deacetylases on N^6^-formyllysine. (A) TK6 cells were treated with the class I and class II histone deacetylase inhibitor, SAHA, as described in *Materials and Methods*. Data represent mean ± SD for N = 3, with asterisks denoting statistically significant differences by Student's t-test (p<0.05). (B) Treatment of peptide substrates containing N^6^-acetyllysine or N^6^-formyllysine with the class III histone deacetylase, SIRT1.

The chemical similarity of N^6^-formyllysine and N^6^-acetyllysine suggested that the former might be subject to removal by lysine deacetylases that recognize and remove N^6^-acetyllysine from histone and other proteins [16], [27]–[30]. Lysine deacetylases fall into several classes, including classes I and II that share a common hydrolytic mechanism and are all inhibited by suberoylanilidehydroxamic acid (SAHA), and the class III enzymes (*i.e.*, sirtuins) that are NAD^+^-dependent deacetylases [31], [32]. In order to assess the activity of lysine deacetylases with N^6^-formyllysine substrates, TK6 cells were treated with SAHA and the levels of N^6^-acetyllysine and N^6^-formyllysine were quantified. The results shown in Figure 6A reveal that, while SAHA caused a 3-fold increase in the level of N^6^-acetyllysine (4 to 14 per 10^3^ lysines), lysine formylation was not affected. To assess sirtuin activity against N^6^-formyllysine, we performed *in vitro* reactions of SIRT1 with a consensus peptide (GGAKRHR) containing N^6^-formyllysine or N^6^-acetyllysine, and the quantities of the modified and unmodified peptides were analyzed by LC-MS/MS. As shown in Figure 6B, SIRT1 removed the acetyl modification completely to generate the unmodified peptide, while only ∼10% (±4%) of N^6^-formyllysine was removed.

## Discussion

N^6^-Formyllysine was first reported in 1985 in reactions of lysine with formaldehyde *in vitro* [33] and, more recently, it was shown to form during *in vitro* silver-staining procedures that involve the use of formaldehyde [34]. The recent discovery of N^6^-formyllysine as a relatively abundant endogenous posttranslational modification of histones and other nuclear proteins in cells [9]–[11] has raised questions about its mechanism of formation and its potential for interfering with the regulatory function of lysine N^6^-acetylation. With respect to formation, we previously presented evidence that N^6^-formyllysine in histones could arise from reactions with 3′-formylphosphate residues derived from DNA oxidation [9]. However, formaldehyde emerged as an alternative source given the high reactivity of formaldehyde toward primary amines, such as the side chain of lysine, and the potential for endogenous oxidation to convert a formaldehyde-derived carbinolamine to a stable formamide (Figure 1). We have now demonstrated *in vitro* and in cells that formaldehyde exposure leads to formation of N^6^-formyllysine residues in proteins. The fact that this modification arises in proteins other than chromatin proteins and in cellular compartments other than the nucleus (Table 2) suggests that 3′-formylphosphate residues in oxidized DNA do not account for all N^6^-formyllysine adducts. This is consistent with recent studies in which N^6^-formyllysine was detected in a protein oxidized with peroxynitrite *in vitro* [5]. The absence of detectable N^6^-formyllysine arising from demethylation of N^6^-methyllysine species (Figure 5C and Figure S2) suggests that interruption of histone demethylation reactions to form the carbinolamine precursor of N^6^-formyllysine occurs at low frequency, or that the formaldehyde produced by complete lysine demethylation [23] does not occur at concentrations high enough to drive formylation of lysine or cause substantial changes in N^6^-formyllysine levels detected by our current analytical method. Furthermore, there is the possibility that the formaldehyde released during lysine demethylation may be scavenged before it could react with lysines in histones. A recent study reports that lysine-specific demethylase 1 (LSD1) is a folate binding protein [35], which led the authors to hypothesize that the biological function of folate is to trap the formaldehyde that is generated during lysine demethylation [35]. In addition, the observation that the formaldehyde equivalent derived from histone demethylation might not account for a significant portion of formyllysine residues is not surprising in light of the abundance of formaldehyde from other cellular processes, such as nucleic acid demethylation and biosynthesis of purines, thymidine and some amino acids [20], [22], [23]. This is clear from the high steady-state concentrations of formaldehyde in human plasma (13–97 µM) [20], though the contribution of long-term, low-level exposure from environmental sources of formaldehyde cannot be ruled out.

**Figure 7:**
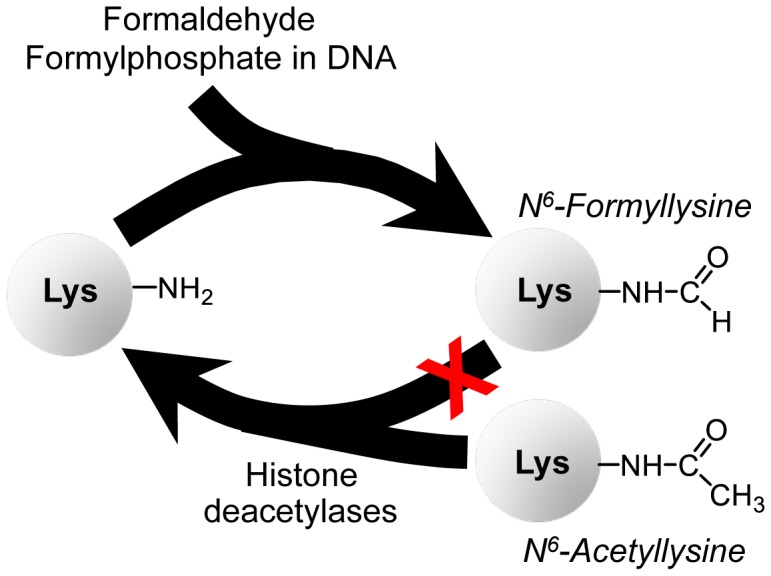
Summary of findings on N^6^-formyllysine in histones. N^6^-formyllysine can arise from reaction of lysine with the 3′-formyl phosphate residue derived from 5′-oxidation of 2-deoxyribose in DNA or from reaction of lysine with formaldehyde. Furthermore, our data suggest that N^6^-formyllysine is refractory to removal by histone deacetylases, which is consistent with the persistence of this pathological adduct throughout the life of individual histone proteins.

The relative abundance of N^6^-formyllysine in histone and other proteins (Table 1 and Table 2) [9]–[11] and the persistence of these adducts in histone proteins provides insights into both their source and their potential effects on cell function. The N^6^-formyllysine residues are relatively evenly distributed among different classes of histone proteins (Table 1), while the other functional modifications show very biased distributions over a large frequency range, which is consistent with the known function and conserved locations for lysine methylation and acetylation [16]–[18]. This random distribution of formyllysine adducts in histone proteins suggests that they are adventitious and not physiological. The fact that N^6^-formyllysine levels are similar in histone and non-nuclear proteins and in all cell compartments also suggests that the sources of this protein modification are equally balanced in the various compartments and proteins, or that there is a single dominant source that distributes uniformly throughout the cell. With regard to its persistence in cells, there is still no evidence supporting the enzymatic removal of N^6^-formyllysine. Our investigation reveals that N^6^-formyllysine adducts appear to be refractory to removal by histone deacetylase enzymes, which suggests that they will persist throughout the lifetime of individual histone proteins. Although our results are consistent with lysine N^6^-formylation as a stable protein modification, we cannot rule out the possibility of an enzyme that removes this modification from selected conserved lysine sites in histone proteins, resulting in small changes in the population level of formyllysine that are not detectable by our current analytical method. Figure 7 summarizes our findings presented here on N^6^-formyllysine adducts.

The high abundance of lysine N^6^-formylation in histone proteins (Table 1) as well as its occurrence at many of the known conserved functional locations for lysine acetylation and methylation in histones [10], [11] suggests that N^6^-formyllysine could interfere with signaling processes associated with physiological histone modifications [16], [27]. Mann and co-workers found formylation of core and linker histones at multiple lysines in both the N- and C-terminal tails and in the globular domains [10], which is consistent with our observation of a random distribution of N^6^-formyllysines among the different histone classes. Several of the formylated lysines occurred at functionally important sites. For example, the observed N^6^-formylation of lysine 12 in histone H4 could interfere with BRD2 bromodomain-dependent transcriptional activation that occurs when the lysine is acetylated [10]. Furthermore, formylation was observed at several lysines known to make contact with the DNA backbone in nucleosomes, which could interfere with nucleosome organization given the conserved acetylation and methylation at these sites [10]. Our observation that N^6^-formyllysine is refractory to removal by several histone deacetylases suggests that the adducts could interfere with the epigenetic regulatory processes mediated by acetylation and methylation. While this potential epigenetic mechanism of disruption of cell function may contribute to the toxicity and carcinogenicity associated with formaldehyde [20], [36]–[40], the association of N^6^-formyllysine with a variety of different cell and organismal processes, including metabolism and the oxidative and nitrosative stresses of inflammation [5], [9], suggests that this adduct may play a role in many pathophysiological processes in humans.

## Materials and Methods

### Materials

Unlabeled and [^13^C,^2^H_2_]-labeled formaldehyde were purchased as 37% and 20% aqueous solutions from Amresco (Solon, OH) and Isotec (Miamisburg, OH), respectively. 4,4,5,5-[^2^H]-Lysine was purchased from Cambridge Isotope Laboratories (Andover, MA). 4,4,5,5-[^2^H]-N^6^-Formyllysine was synthesized from 4,4,5,5-[^2^H]-lysine according to Jiang *et al.* [9]. 3,3,4,4,5,5,6,6-[^2^H]-N^6^-Acetyllysine were obtained from CDN Isotopes (Pointe-Claire, Quebec, Canada). L-Methionine-([^13^C,^2^H_3_]-methyl) was obtained from Isotec (Miamisburg, OH). L-Lysine, N^6^-formyllysine, N^6^-acetyllysine, bovine serum albumin, human recombinant HMG-1, human IgG, *Streptomyces griseus* protease, and protease inhibitor cocktail (for use with mammalian cell and tissue extracts) were obtained from Sigma-Aldrich (St. Louis, MO). N^6^-Mono-methyllysine, N^6^-di-methyllysine, and N^6^-tri-methyllysine were purchased from Bachem Bioscience Inc. (King of Prussia, PA). Nonidet P-40 was from Roche Diagnostic Corporation (Indianapolis, IN). Suberoylanilidehydroxamic acid (SAHA) and SIRT1 (human recombinant) enzyme were purchased from Cayman chemical (Ann Arbor, MI). Peptide substrates for SIRT1 (GGAKRHR and its lysine-acetylated and -formylated forms) were synthesized at Massachusetts Institute of Technology Biopolymers Laboratory. The human lymphoblastoid TK6 cell line was a generous gift of Prof. Gerald Wogan (Massachusetts Institute of Technology).

### TK6 cell culture, exposure, and labeling

TK6 cells were cultured in RPMI 1640 medium (Cellgro, Manassas, VA) supplemented with 10% heat-inactivated horse serum (Atlanta Biologicals, Lawrenceville, GA), 10,000 U penicillin/ml and 10,000 µg streptomycin/ml (Lonza, Walkersville, MD), and 2 mM L-glutamine (Lonza, Walkersville, MD) at 37°C in a 5% CO_2_ atmosphere. For formaldehyde exposure studies, TK6 cells were pelleted, washed, and resuspended in RPMI 1640 medium without any supplements, prior to addition of formaldehyde to the medium. Following addition of formaldehyde, cells were incubated at 37°C for 2 h with occasional mixing prior to extracting chromatin proteins. Histones were extracted from ∼10^7^ cells after exposure and the quantity of formyllysine, as a percentage of total lysine, was measured as described below. For lysine demethylation studies, TK6 cells were grown in a customized RPMI-1640 medium identical to the traditional medium (*e.g.*, supplemented with horse serum, antibiotics, and L-glutamine), except for the presence of labeled methionine (L-methionine-([^13^C,^2^H_3_]-methyl)) instead of non-labeled methionine. Histones (from ∼10^7^ cells) were extracted every 2 d for 20 d in order to investigate the formation of N^6^-[^13^C,^2^H]-formyllysine. For histone deacetylase studies, TK6 cells were incubated with the histone deacetylase inhibitor, suberoylanilidehydroxamic acid (SAHA), for 20 h at 37°C in a 5% CO_2_ atmosphere prior to histone extraction. SAHA was dissolved in a 50∶50 solution of DMSO∶PBS prior to addition to cell media. Control cells (∼10^7^) were treated with the DMSO∶PBS vehicle.

### Histone extraction from TK6 cells and subcellular protein fractionation from tissue

Extraction of histones was performed according to Boyne *et al.* [41], with modifications. Cells (∼10^7^ per sample) were pelleted by centrifugation at 1000× g for 5 min at 4°C and the pellet was washed once with PBS. Cell pellets were then lysed by resuspension in ice-cold lysis buffer (15 mM Tris-HCl, pH 7.5, 15 mM NaCl, 60 mM KCl, 1 mM CaCl_2_, 5 mM MgCl_2_, 250 mM sucrose, 1 mM dithiothreitol, 10 mM sodium butyrate) containing a 100∶1 v∶v dilution of protease inhibitor cocktail in the presence of 0.03% Nonidet P-40 and incubation on ice for 5 min with occasional gentle mixing. Nuclei were pelleted by centrifugation at 600× g for 5 min at 4°C, and the pellet was washed twice with ice-cold lysis buffer without Nonidet P-40. Histones were extracted by addition of ice-cold 0.4 N H_2_SO_4_ and incubation overnight on ice. The solution was centrifuged at 3000× g for 5 min and proteins in supernatant were precipitated by addition of 20% v/v trichloroacetic acid and overnight incubation at 4°C. Samples were then centrifuged at 14000× g for 10 min at 4°C, washed once with ice-cold acetone containing 0.1% HCl, and once with ice-cold acetone. The extracts were air-dried and stored at −20°C until use. For collecting membrane, cytosolic, and nuclear fractions, 20 mg of bovine liver tissue was cut into small pieces and washed with PBS, and proteins were fractionated using the Subcellular Protein Fractionation Kit from Thermo Scientific (Waltham, MA) and a Kontes all-glass Dounce homogenizer (10 strokes with a type B pestle). Proteins in subcellular extracts were precipitated by addition of 20% v/v trichloroacetic acid and overnight incubation at 4°C. Samples were then centrifuged at 14000× g for 10 min at 4°C, washed once with ice-cold acetone containing 0.1% HCl, and once with ice-cold acetone. The extracts were air-dried and stored at −20°C until use.

### Purification of individual histones

HPLC purification of total histones was performed according to Boyne *et al.* [41] with modifications. Total histones (≤50 µg) were dissolved in 0.1% trifluoroacetic acid (TFA) in water and fractionated by HPLC on an Agilent 1100 series instrument (Agilent Technologies, Santa Clara, CA), using a Source 5RPC C_18_ reversed-phase column (4.6×150 mm, 5 µm particles; GE Healthcare Life Sciences). The mobile phase flow rate was 1 mL/min and the solvent system was 0.1% TFA in water (A) and 0.094% TFA in acetonitrile (B) with the elution starting at 0% B, linearly increasing to 28% B over 28 min, reaching 37% B at 70 min, 38% B at 100 min, 60% B at 150 min, and finally 100% B at 151 min, before the column was re-equilibrated to 0% B for 10 min. Protein elution was monitored by UV absorbance at 214 nm and histones in each fraction were tentatively identified by resolution on a 13% SDS-PAGE gel with Coomassie Blue staining (see Figure S1).

### Enzymatic hydrolysis of proteins

Histones extracted from TK6 cells and other protein samples were dissolved in 50 µL of 100 mM ammonium bicarbonate buffer (pH 8.5), 4,4,5,5-[^2^H]-lysine (2 nmol),4,4,5,5-[^2^H]-N^6^-formyllysine (1 pmol), and 3,3,4,4,5,5,6,6-[^2^H]-N^6^-acetyl lysine (10 pmol) were added as internal standards, and the proteins hydrolyzed by addition of *S. griseus* protease (freshly prepared solution each time) with incubation at 37°C for ≥16 h. *Streptomyces griseus* was used at a ratio of 1 µg of enzyme per 10 µg of protein. Samples were then dried under vacuum and resuspended in 50 µL of water before mass spectrometry analysis.

### Quantification of amino acids

N^6^-Formyllysine and other amino acids were quantified as a percentage of the total quantity of lysine, by liquid chromatography-coupled mass spectrometry (LC-MS/MS). HPLC was performed with an Agilent 1100 series instrument. Adducts of interest in the resuspended protein hydrolysates were separated using an aqueous normal phase Cogent diamond hydride column (2.1×150 mm, 4 µm) from MicroSolv Technology Corporation (Eatontown, NJ). The mobile phase flow rate was 400 µL/min, and the column temperature was maintained at 20°C. The solvent system was 0.1% acetic acid in water (A) and 0.1% acetic acid in acetonitrile (B), with the elution starting at 100% B, the gradient linearly decreased to 25% B over 30 min, stayed at 25% B for 3 additional min before the column was re-equilibrated at 100% B for 7 min. In order to separate a co-eluting contaminant from pre-purified lysine demethylation study samples, an extended chromatography run time was used, with the elution starting at 100% B, the gradient linearly decreased to 75% B over 75 min, further decreased to 25% B over the next 3 min, reached 15% by 83 min before the column was re-equilibrated at 100% B for 7 min. The species of interest were then analyzed using Agilent 6410 triple quadrupole mass spectrometer (MS/MS) equipped with an electrospray ionization (ESI) source operated in positive ion mode. The operating parameters were as follows: ESI capillary voltage, 4000 V; gas temperature, 350°C; drying gas flow, 12 L/min; and nebulizer pressure, 30 psi. Selected reaction monitoring (SRM) transitions are summarized in Table 3. Note that in addition to chromatographic separation (Figure 2) and presence of internal standards, the unique product ions of 112 *m/z* and 126 *m/z* for formyl and acetyl lysines, respectively, distinguish them from their isobaric compounds di- and tri-methyl lysines. The fragmentor voltage and collision energy were optimized in order to maximize the signal of each product ion monitored (see Figure S3) and are summarized in Table 3. 4,4,5,5-[^2^H]-Lysine, 4,4,5,5-[^2^H]-N^6^-formyllysine, and 3,3,4,4,5,5,6,6-[^2^H]-N^6^-acetyl lysine were used as internal standards. Calibration curves for the labeled and unlabeled forms of these analytes were constructed by plotting the ratios of the areas of the MS signals for the labeled and unlabeled forms against their corresponding concentration ratios (Figure S4). N^6^-Methylated lysine species were quantified using the deuterated acetyl lysine internal standard signal (Figure S4).

**Table 3:** Summary of mass spectrometry parameters for each species.

Species of Interest	SRM Transition (m/z)	Internal Standard Transition (m/z)	Qualifier Ion Transition (m/z)	Fragmentor Voltage (V)	Collision Energy (V)
Lysine	147→130	151→134	NA	100	8
Formyllysine	175→112	179→116	NA	105	10
Acetyllysine	189→126	197→134	NA	105	10
Mono-methyllysine	161→84	NA	161→130	100	10
Di-methyllysine	175→84	NA	175→130	100	14
Tri-methyllysine	189→84	NA	189→130	100	14

### SIRT1 peptide experiment

SIRT1 peptide substrates (GGAKRHR, GGAK_acetyl_RHR, and GGAK_formyl_RHR) were HPLC purified on an Agilent 1100 series instrument using Vydac 218TP52 C18 reverse-phase column (2.1×250 mm, 5 µm) from Grace Vydac (Hesperia, CA). The mobile phase flow rate was 200 µL/min, and the column temperature was maintained at 30°C. The solvent system was 0.05% trifluoroacetic acid in water (A) and 0.05% trifluoroacetic acid in 80% acetonitrile (B), with the isocratic elution of 5% B for the first 5 min, then a linear increase to 42% B over 25 min, reaching 100% B at 31 min followed by column re-equilibration at 5% B for 10 min. Each purified SIRT1 peptide substrate (100 pmol) were incubated overnight (12 h) at 25°C with 1 µg SIRT1, in 50 mM Tris-HCl (pH 8) buffer containing 137 mM NaCl, 2.7 mM KCl, 1 mM MgCl_2_, and 6 mM NAD^+^. The removal of acetyl and formyl groups from SIRT1 peptide substrates was determined using liquid chromatography-coupled mass spectrometry. HPLC was performed on an Agilent 1100 series instrument using Agilent Exclipse XDB C18 reverse-phase column (4.6×150 mm, 5 µm). The mobile phase flow rate was 200 µL/min, and the column temperature was maintained at 40°C. The solvent system was 0.1% acetic acid in water (A) and 0.1% acetic acid in acetonitrile (B), with the elution starting at 20% B, the gradient linearly increased to 50% B over 5 min, reached 100% B at 6 min, and kept at 100% B for 9 minutes before the column was re-equilibrated at 20% B for 10 min. The species of interest were then analyzed using the Agilent 6410 MS/MS system equipped with an electrospray ionization (ESI) source operated in positive ion mode. The operating parameters were as follows: ESI capillary voltage, 3500 V; gas temperature, 345°C; drying gas flow, 8 L/min; and nebulizer pressure, 30 psi. Multiple reaction monitoring (MRM) transitions were as follow: GGAKRHR peptide, *m/z* 781.1→625.2; GGAK_formyl_RHR peptide, *m/z* 809.4→516.3; and GGAK_acetyl_RHR peptide, *m/z* 823.4→530.4. The fragmentor voltage and collision energy were 200 V and 40 V for GGAKRHR peptide, respectively; 100 V and 46 V for GGAK_formyl_RHR peptide; and 100 V and 52 V for GGAK_acetyl_RHR peptide.

## Supporting Information

Figure S1 Reversed-phase HPLC fractionation of histone proteins. (A) HPLC elution profile for histones extracted from TK6 cells, as described in *Materials and Methods*. (B) SDS/PAGE analysis of the HPLC-fractionated proteins shown in panel A. Lanes 1 and 2 are molecular weight markers, while lanes 3 and 4 refer to total histones from TK6 cells and calf thymus, respectively.
(TIF)

Figure S2 Lysine demethylation is not a source of N^6^-formyllysine in histones. By culturing TK6 cells in customized RPMI medium containing L-Methionine-([^13^C,^2^H_3_]-methyl) for 20 days, it was shown that in contrast to predominant heavy isotope labeling of mono-methyllysines (>90%), even as early as day 2, the level of N^6^-[^13^C, ^2^H]-formyllysine did not show an increase beyond the natural isotope abundance level (∼0.7% for [M+2] ion of N^6^-formyllysine), for any day.
(TIF)

Figure S3 Examples of product ions for each lysine species monitored, after optimization. In all cases, the highest count was used as the product ion for MRM in triple quadruple mass spectrometer, as described in *Materials and Methods*. An exception was lysine, for which the 130 *m/z* ion was selected due to lysine's high abundance compared to other species monitored.
(TIF)

Figure S4 Examples of calibration curves for the isotope-dilution LC-MS/MS analysis of modified lysine species, as described in *Materials and Methods*. Abbreviations: FK, N^6^-formyllysine; DFK, deuterium-labeled N^6^-formyllysine; AK, N^6^-acetyllysine; DAK, deuterium-labeled N^6^-acetyllysine; K, lysine; DK, deuterium-labeled lysine; MK, N^6^-mono-methyllysine; M_2_K, N^6^-di-methyllysine; M_3_K, N^6^-tri-methyllysine.
(TIF)
