# Supplementary figures and images for: Quantitative Analysis of Histone Modifications: Formaldehyde Is a Source of Pathological N6-Formyllysine That Is Refractory to Histone Deacetylases
Source: PLoS Genet. 2013 Feb 28;9(2):e1003328. doi: 10.1371/journal.pgen.1003328 (PMC3585032; doi:10.1371/journal.pgen.1003328)

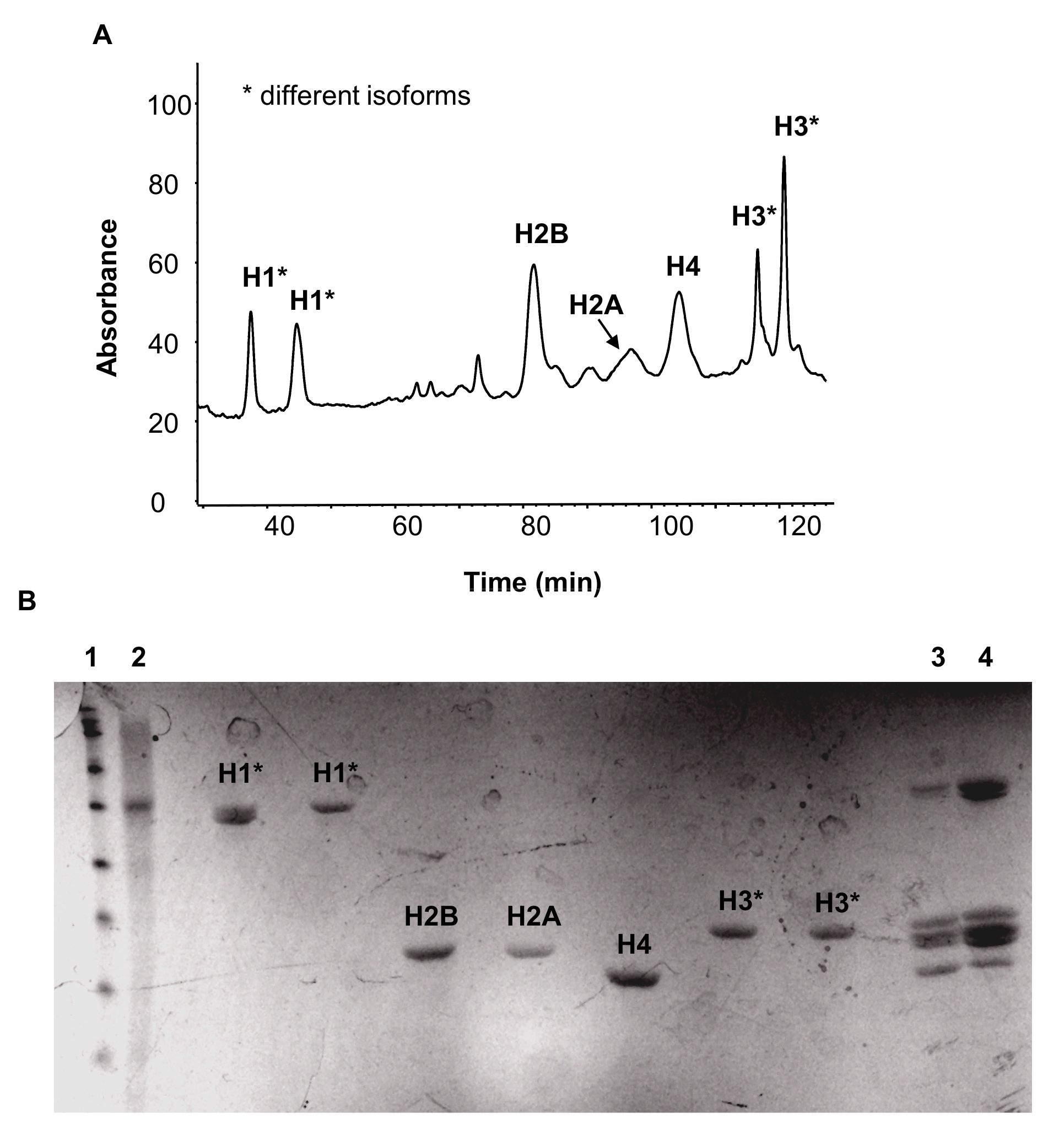

Supplement: Figure S1 — Reversed-phase HPLC fractionation of histone proteins. (A) HPLC elution profile for histones extracted from TK6 cells, as described in Materials and Methods . (B) SDS/PAGE analysis of the HPLC-fractionated proteins shown in panel A. Lanes 1 and 2 are molecular weight markers, while lanes 3 and 4 refer to total histones from TK6 cells and calf thymus, respectively. (TIF) [file pgen.1003328.s001.tif]

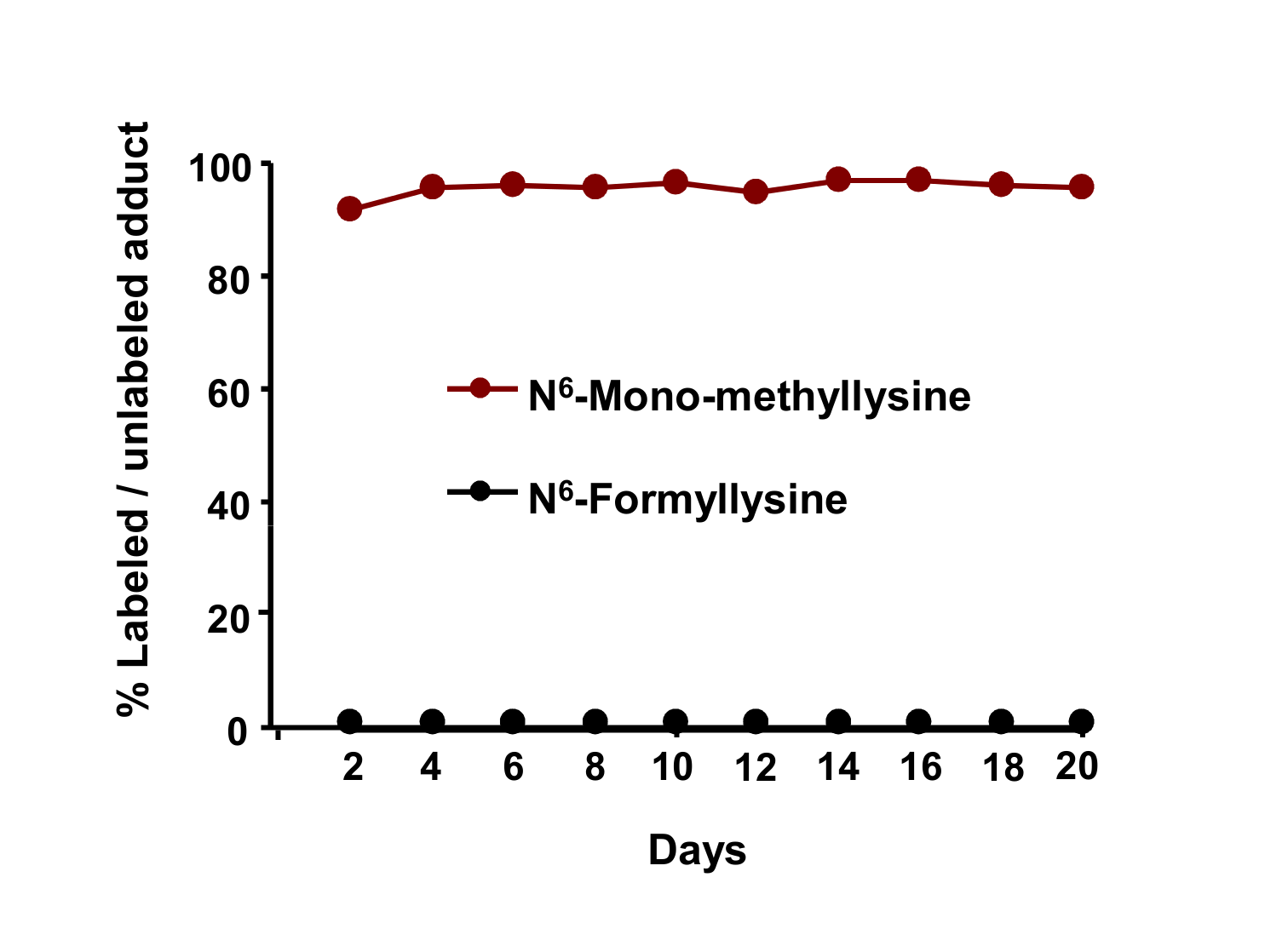

Supplement: Figure S2 — Lysine demethylation is not a source of N6-formyllysine in histones. By culturing TK6 cells in customized RPMI medium containing L-Methionine-([13C,2H3]-methyl) for 20 days, it was shown that in contrast to predominant heavy isotope labeling of mono-methyllysines (>90%), even as early as day 2, the level of N6-[13C, 2H]-formyllysine did not show an increase beyond the natural isotope abundance level (∼0.7% for [M+2] ion of N6-formyllysine), for any day. (TIF) [file pgen.1003328.s002.tif]

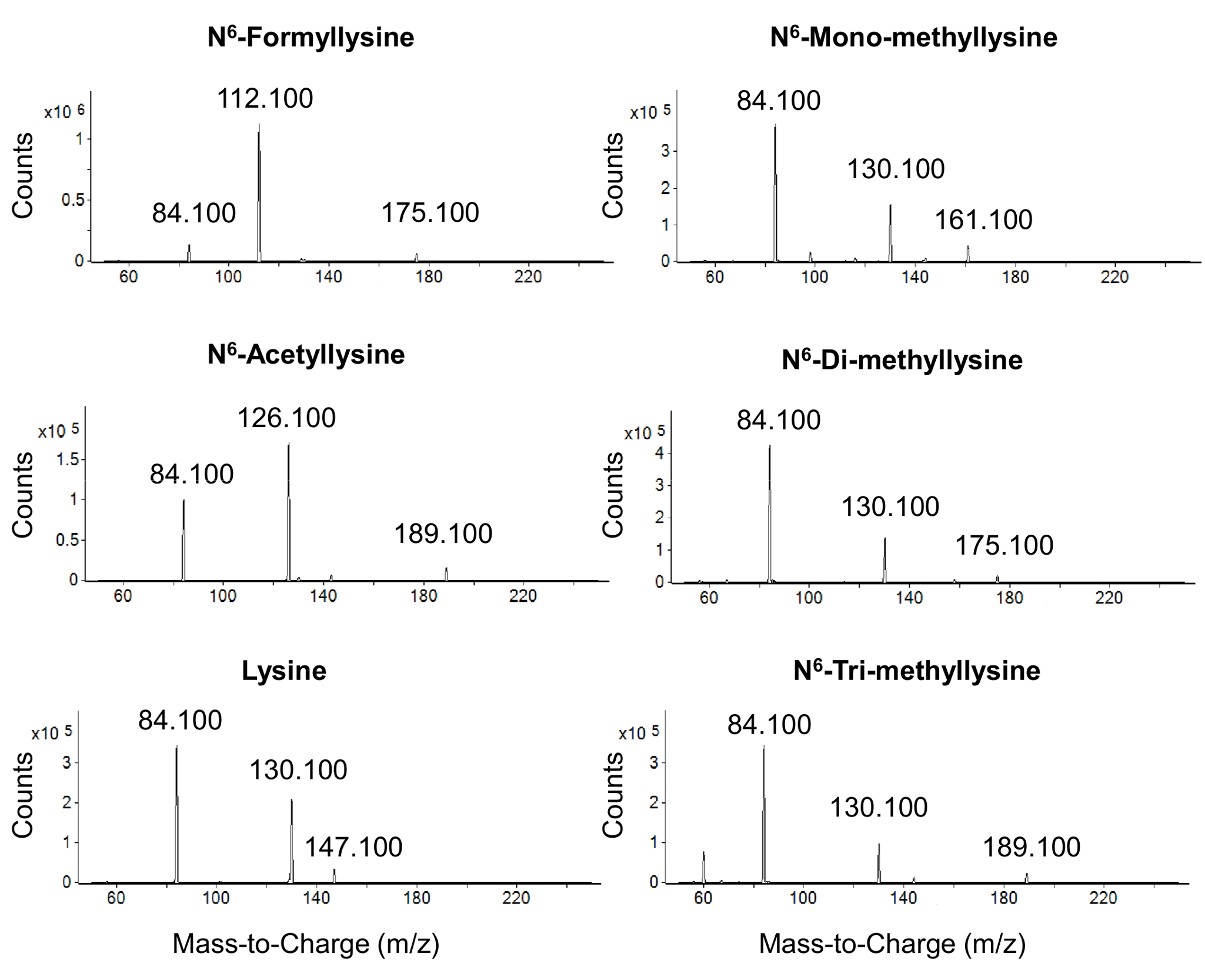

Supplement: Figure S3 — Examples of product ions for each lysine species monitored, after optimization. In all cases, the highest count was used as the product ion for MRM in triple quadruple mass spectrometer, as described in Materials and Methods . An exception was lysine, for which the 130 m/z ion was selected due to lysine's high abundance compared to other species monitored. (TIF) [file pgen.1003328.s003.tif]

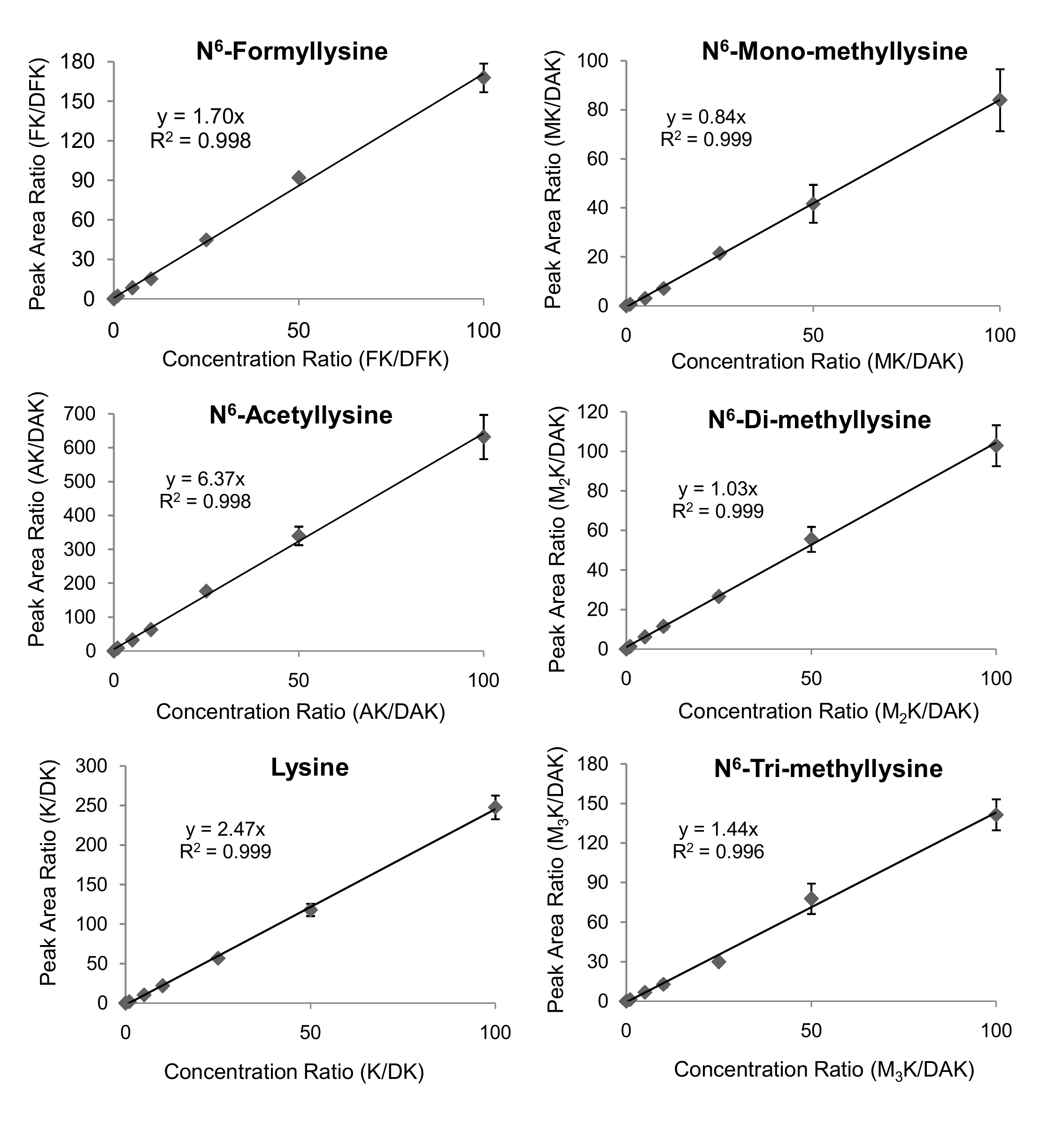

Supplement: Figure S4 — Examples of calibration curves for the isotope-dilution LC-MS/MS analysis of modified lysine species, as described in Materials and Methods . Abbreviations: FK, N6-formyllysine; DFK, deuterium-labeled N6-formyllysine; AK, N6-acetyllysine; DAK, deuterium-labeled N6-acetyllysine; K, lysine; DK, deuterium-labeled lysine; MK, N6-mono-methyllysine; M2K, N6-di-methyllysine; M3K, N6-tri-methyllysine. (TIF) [file pgen.1003328.s004.tif]
